# Human Responses and Adaptation in a Changing Climate: A Framework Integrating Biological, Psychological, and Behavioural Aspects

**DOI:** 10.3390/life11090895

**Published:** 2021-08-29

**Authors:** Paolo Cianconi, Batul Hanife, Francesco Grillo, Kai Zhang, Luigi Janiri

**Affiliations:** 1 Department of Neurosciences, Section of Psychiatry, Catholic University, 00168 Rome, Italy; luigi.janiri@unicatt.it; 2 Provincial Agency for Health Services, Institute of the Autonomous Province of Trento, 380123 Trento, Italy; batul.hanife@gmail.com; 3 Department of History, Anthropology, Religions, Art History, Media and Performing Arts, Sapienza University of Rome, 00185 Rome, Italy; franpaog@yahoo.it; 4 Department of Environmental Health Sciences, School of Public Health, University at Albany–State University of New York, Rensselaer, NY 12144, USA; kzhang9@albany.edu

**Keywords:** climate change, vulnerability, resilience, mental maladjustment, emergent behaviour, mass effect phenomena

## Abstract

Climate change is one of the biggest challenges of our times. Its impact on human populations is not yet completely understood. Many studies have focused on single aspects with contradictory observations. However, climate change is a complex phenomenon that cannot be adequately addressed from a single discipline’s perspective. Hence, we propose a comprehensive conceptual framework on the relationships between climate change and human responses. This framework includes biological, psychological, and behavioural aspects and provides a multidisciplinary overview and critical information for focused interventions. The role of tipping points and regime shifts is explored, and a historical perspective is presented to describe the relationship between climate evolution and socio-cultural crisis. Vulnerability, resilience, and adaptation are analysed from an individual and a community point of view. Finally, emergent behaviours and mass effect phenomena are examined that account for mental maladjustment and conflicts.

## 1. Introduction

Climate change has continuously affected our planet in the past causing biodiversity losses, collapses, or reshaping of societies and cultures, and it requires long periods for recovery and return to new “normal”, albeit different, balances. In recent years, it has been considered an urgent economic, social, and existential threat worldwide, so increasing attention has been directed to implement appropriate measures to prevent and/or avoid consequential catastrophes [1,2]. Its effects are exponentially increasing, but the ultimate speed of current change is not determined [3]. Instability, climatic variability, rapid, and abrupt transitions can lead to more frequent and intense extreme weather events and to dangerous consequences [4] and even changes in our biosphere, of which the effects are still unclear [5]. Climate change hits rigid systems [6] or very specialized species as well as everything that has been already weakened by other factors (e.g., genetics, demography, food). During transitions, species that were fit may become vulnerable, other species may adopt changes appropriate for resilience, while other species may initially succeed and weaken later on [7,8], including humans. There exist short- and long-term strategies to respond: the first include migration to more fitted and more advantageous environments, the second are genetic and phenotypic/epigenetic adaptations [9]. Moreover, humans can also modify the environment using technology. Unfortunately, there are no reliable indicators and tools to predict responses and evolutionary processes in such conditions and on a global scale [8,10], and it is unknown how to maintain the fitness of species in such environmental heterogeneity. Moreover, among the population there is still a meagre awareness and limited information concerning the severity of the climate change and its consequences, which can be abstract for individuals [11,12]. Sometimes people may show cognitive and emotional resistance or paradoxical behaviours [13,14,15], in spite of the frequency of extreme weather events, with perhaps the naïve confidence that human technology might be able to resolve all issues.

Research models are trying to deepen the consequences of climate change and its impact on both the animal and human population, allowing a deeper comprehension of some topics. Nevertheless, many studies have focused on single aspects opening a debate relating to contradictory observations, and they often lack in contextualizing them in the processes of charge over time within the history of Earth. Moreover, climate change is a complex phenomenon with entanglements of biophysical and socio-political systems and cannot be addressed adequately from the perspective of a single discipline.

To address these gaps, we propose a conceptual framework on the relation between climate change and human responses. We present the more relevant information integrating biological, psychological, behavioural, and social aspects and their interdependencies, in order to obtain a complete and consistent overview on the issue from a multidisciplinary point of view.

### 1.1. Climate Change

Scientists are monitoring several phenomena associated with climate change, but its consequences are often not clear at the very beginning, and not all of them are equally relevant to human adaptation [11,12]. In our perspective, the following are the most important definitions to bear in mind.

Extreme climate events (ECEs): they are the consequences induced by climate change (e.g., floods, droughts, heat waves, storms). Unlike natural disasters, they are not occasional events in a stable system, whereas they are more frequent and extreme and long-lasting. However, there is still no consensus on the definition, since it is not clear how to classify their extremes, frequency, and impacts [16]. In this article, we do not consider planetary-scale extreme events (PEEs), global catastrophes that are principally physical (such as chain of major volcano eruption or meteorite impact) causing planetary climatic or atmospheric regime shifts and mass organismal extinction and driving macro-evolution [16].

Tipping point: This is the process of reaching a critical threshold that compromises the state of a system [17]. Most of the studies on climate agree that changes may occur gradually, and indeed, climate change still shows sporadic extreme climatic events instead of a clear widespread global disaster, but abrupt changes may also occur [18]. The multiplicity of variables involved [19] and the relative stability near a tipping point can overshadow the true meaning of these early warning signs [20], even if the whole system is on the brink of collapse. Tipping points are self-reinforcing and interact with other systems triggering other tipping points, thus leading to a large shift that might be difficult to reverse [21]. Critical situations induced by climate change can be considered a tipping point especially in rigid or already weakened systems [6]. Several tipping points are currently active (such as deforestation, Arctic ice retreating, loss of biodiversity) and other potential ones have already been identified [22].

**Figure 1:**
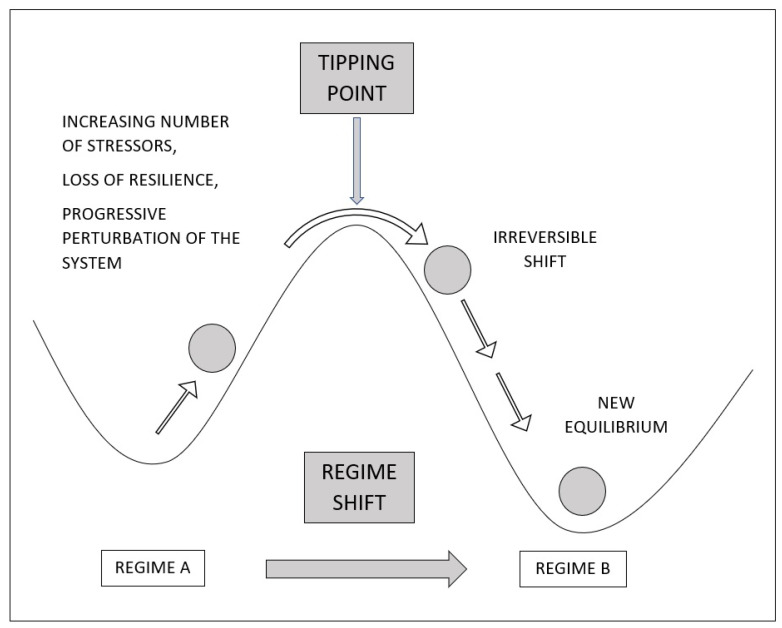
The increasing number of stressors can cause the loss of resilience and the perturbation of the system, reaching a critical threshold (tipping point) that compromises the current state and leads to an irreversible regime shift to a new equilibrium (from regime A to regime B).

Regime shift: In climate systems, it is the global change of the climate when different activated tipping points can overcome the actual climate equilibrium (Figure 1). It is hard to detect whether and if a system is in the proximity of a tipping point [23]; sometimes, it can still return back into a normal range. Nonetheless, those systems become very slow in recovering from perturbations (a phenomenon known as “critical slowing down”) [7]. Moreover, when a system is unstable, even a small perturbation can trigger a cascade of events leading the whole system to disruption or to a massive shift. In some cases, the instant effects are smaller and even unnoticed, while the irreversible damage is revealed later on by other extreme events [24], which is mainly the case of present climate change. However, when a new climate system is reached it will resist further changes: because of global warming, the thermal inertia of the oceans, and the carbon cycle, the regional temperature will remain high long after emissions have ceased and CO2 concentrations in the atmosphere eventually decrease (and some models suggest a 1000 years’ timescale) [25].

Human activities are triggering biosphere tipping points, which in turn can activate a chain of events up to regime shift that can lead to the so-called “Hothouse Earth” [26]. It is not known exactly to what extent human activities will influence the climate; however, the predictions are based on accurate data and modelling. The increase in atmospheric CO₂ concentrations, from a relatively stable value of 280 ppm (parts per million) to the actual 414 ppm in the last two centuries [6,27], has never been so high in 800,000 years [3]. Paleoclimate researchers are stressing that new simulation would indicate that CO₂ levels could reach levels comparable to those more than 50 million years ago of the Eocene epoch (1000 ppm by the year 2100) [28]. This is leading to other phenomena that are potentially catastrophic causing a further worsening of global warming, e.g., atmospheric heating, ice melting in the Arctic and Antarctic, permafrost melting with the release of greenhouse gas, glaciers melting with floods, oceans heating with acidification, and an increase in wildfires. This in turn might lead to the worsening of the direct impact of human activities, such as deforestation, intensive agriculture, water exploitation, and pollution. The combination of these phenomena can cause the instability of the climate system, reaching tipping points and regime shifts. It worth noting that, in the past, the severity of changes in climate that lasted years or decades was limited and local, while the more extreme and global changes in the climate lasted much longer. On the contrary, we are now facing changes that are short-term, extreme, and global at the same time [29]. In the future, the climate will possibly reach a new feasible equilibrium, but the major risks are during this dangerous transition period, since our civilization is built on the previous equilibrium.

### 1.2. The Historical Perspective: What Can We Learn from the Past?

The historical point of view offers empirical data and considerable clues, such as what to monitor and how to set a model. Moreover, it illustrates the concrete evidence of future risk and may support a more effective mass communication. In fact, natural variations in the Earth’s climate have accompanied humanity from its origins, and catastrophic climatic changes have played an important role in the evolution of our species [30]. Numerous historical examples show that climate crises, especially in cases of sudden climate shifts, produced or have been associated with severe social and cultural crises (Table 1). Some of these events are documented by historical documents, while others are still not confirmed hypotheses [31,32]. Causes can vary (e.g., loss of resources, agricultural crisis, epidemic), but the most dangerous threats are those on the means of support and human capital, which must be considered warning signs and predictive parameters. Attention is to be paid to the potential risk of isolation. Rigid, hierarchical, and centralized societies are more likely to collapse, whereas nomadic populations usually migrate. However, sometimes a different social, political, and cultural system can develop, also setting up a complex civilization.

For instance, in the Middle East, after the end of the Ice Age, the culture of the Natufian villages spread until the arrival of the cold Dryas period causing the disappearance of their settlements [30]; whereas, later on, a new thermal optimum probably helped the Neolithic agricultural revolution. In Mesopotamia, a change in climate on the coastline played a key role in the emergence of city-states after 3500 BC, but after 2150 BC, a drought caused the collapse of the Akkadian empire [30,33]. This drought was probably the same that in Egypt caused the disappearance of the Nile flood with famine and riots and eventually the collapse of the Old Kingdom (around 2159 BC). Egypt was hit again by drought during the Middle Kingdom with similar consequences and, then, followed the invasion of the Asian Hyksos. The same pattern occurred in India with the collapse of the Harappa civilization around 1700 BC followed by the invasion of the Aryans.

The Roman Empire had been also affected by changes in the climate: a hot climate optimum during the 1st century BC might have helped Rome in its expansion to the north, while North Africa was not as dry as today. The end of this climate optimum could have been contributed to upheavals, a crisis in the agricultural system, consequently leading to political, civil, and military instability that also brought about migrations of barbarian peoples starting from the end of the 2nd century AD [34]. Then, from the 5th century AD to the 7th century AD, there is evidence of a climate crisis that could have been one of the concurrent causes of the fall of the Roman western empire [6,30].

In the American continent, it has been suggested that the crisis of the Mayan civilization and its collapse in the 9th century was probably the result of an agricultural crisis caused by a series of heavy droughts draining the aquifers and eroding arable land [35], and the Anasazi pueblos were abandoned at the end of the 13th century almost certainly because of a great drought dated around 1275 [36]. This “Medieval Warm Period” ended around 1300 with a transition to a cold and extremely rainy climate, followed by the “Little Ice Age”.

As we mentioned, changes in the climate with consequences on agriculture and means of subsistence caused in many cases the weakening of the population by malnutrition, with increased mortality risk during epidemics. This is what probably happened in Europe in the year 1347 during the “Black Death” (an epidemic of bubonic plague), resulting in huge devastation, with the resurgence in periods of adverse climate conditions and poor harvests (between 1570 and 1630 in particular) [37], and in China, also in the 14th century, this led to a peasant riot that contributed to driving out the Mongols and the accession of the Ming dynasty [30]. Moreover, famine and pestilence were sometimes interpreted as a divine punishment in mass-social reactions: people were searching for scapegoats, and religious leaders gained more power [30], and that seems to be the case of the peak of witch-hunts throughout the 17th century during the greater climate cooling in Europe.

However, the most severe crisis was in Greenland [35], where the sharp worsening of climatic conditions was the main cause for the disappearance of the Viking colonies at their height after four centuries of existence during the Medieval hot period [30]: the increasing difficulty in maintaining maritime connections resulted in severe isolation from commerce and communications with the Inuit until the colonies disappeared during the 15th century [6,35].

Starting from the 19th century, the impact of human activities on the environment began to be visible: the Industrial Revolution initially enabled mankind to partly free itself from natural catastrophes but caused destructive consequences inducing more influence on climate than natural system oscillations around homeostasis [6].

## 2. Vulnerability and Resilience

In this scenario, humans are facing a potential threat to their existence, especially when a regime shift occurs, since it may abruptly overcome resilience with no chance to reinforce vulnerabilities and avoid the damage. Vulnerability to climate change is defined as “the degree to which a system is susceptible to, or unable to cope with, adverse effects of climate change” [38,39]. Health risks associated with climate change vary depending on multiple factors, such as the nature of the exposure (e.g., the location of a population exposed to drought or flooding), the associated hazards (e.g., projected change in precipitation patterns or climate suitability for infectious disease transmission), socio-economic and environmental determinants for the population and individual (e.g., age, gender, water coverage, sanitation, and hygiene systems), and the capacity of health systems to protect against current and future risks [40,41,42]. Indeed, some populations are disproportionately disadvantaged in dealing with climate change and may more easily develop stress-related psychopathological reactions and/or disorders [39,43,44] due to their geographical position (coasts, areas with intense hurricane activity or subjected to heat waves), to involvement in climate-sensitive activities (agriculture, aquaculture, fishing, pasture) or to reliance on natural resources and ecosystem services for their lives [45,46,47,48,49]. On the other hand, farmers successfully coping with climate change consequences and integrating into a larger economy may nonetheless be affected by market failures caused by unfair and unpredictable patterns of globalized trade and become a vulnerable group [50]. Recognize major vulnerabilities is necessary to identify possible targets and future risks from extreme events [51] and to reduce erroneous predictions and misplaced conservation efforts [52]. In general, poverty is an important condition linked to vulnerability to climate change, as it is directly associated with limited resource access [53], and women, especially in poorer countries such as India, China, and Brazil [54], are the more affected [55,56,57,58]. This can lead to a feminization of poverty and an increasing trend of male out-migration for remittances [59]. Children and the elderly must be considered too: children show vulnerability especially in terms of long-lasting or irreversible outcomes [60,61,62], showing post-traumatic reactive phenomena [63]; during disasters, they can be separated from their families, schools, or childcare centres, and at a later time, it can be harder for them to continue their education [64] or they may show poorer academic performance [65], while elderly people are notoriously attached to their environment and share the same vulnerabilities of individuals who are disabled, chronically ill, or with pre-existing medical conditions [41]. Other vulnerable groups include people with mental illness, homeless, minorities, migrants, and refugees [66] that generally have limited access to resources and lack the adaptive capacity to protect themselves [53], with the risk of negative mental health outcomes [67]. Moreover, it is worth mentioning that many environmental events can cause displacement, but the concept of a climate displaced person is still vague with no clear legal protection [68], so that climate migrants might struggle with isolation, unreliable living and working conditions, or discrimination, as well as frequent physical and mental health problems [69].

Particular attention should be given to indigenous communities because they are exposed to more than one factor of vulnerability [56,70,71] and offer a striking example of coping difficulties [51,53,72]. They are often located in geographically peripheral and vulnerable regions [73] (such as the Arctic [74], but also Africa due to the strong effects of climate change and low adaptive capacity, small islands due to stronger storms and rising sea levels, Asian and African mega deltas, which are densely populated, often with vulnerability to a rise in sea level and low adaptive capacity [68]), several communities face ethnic discrimination, racism, prejudice, bullying, disempowerment [75,76,77], and grief associated with the loss of homelands and their traditional way of life. Vulnerability stems from colonial intrusion into a traditional lifestyle, rapid modernization, and cultural assimilation policies, which cause a loss of traditional knowledge and limit traditional adaptation strategies [78,79]. All these circumstances may be worsened by climate change affecting lifestyle and health, both physical (altering outdoor activities and diet) and mental (inducing stress, worry, depression, anxiety, trauma, and cognitive biases) [74,80], but also resulting in spiritual loss [56] caused by the transformation or the disappearance of sacred ritual important sites and the disrupted relationship with the land [81] that is the source of ancestral linkages [82], even leading to high rates of substance abuse, violence, and suicide [82,83,84].

On the other hand, resilience is defined as the “capacity of a social-ecological system to cope with a hazardous event or disturbance, responding or reorganizing in ways that maintain its essential function, identity, and structure, while also maintaining the capacity for adaptation, learning and transformation” [85], the maximum perturbation a system can tolerate before a transition to an alternative stable state. At an individual level, resilience depends on inherited biological and psychological factors involving personal history, skills, experiences, cognitive and behavioural efforts, social context, and occurrence of adverse events [86] and allows us to cope with adverse events, possibly resist changes, and recover from distress [87]. It has been argued that resilient people anticipate risks, reduce vulnerability to those risks, respond effectively to threats, and recover faster, thus increasing the capacity to respond to subsequent threats [43]. On a social level, resilience is the ability of communities to prevent, recognize, and confront uncertainty [88], it enables adaptive preparation for future events [89], responds to damaging events, mitigates external shocks, deals and manages the changes to infrastructure and the environment, provides aid and services to disaster victims, directs economic and social systems to enable recovery [90]. It is a network created collectively between its components and their adaptive abilities to organize survival in a more complex and uncertain world [91,92]. This perceived social cohesion has been linked to a supportive policy environment at the national and international level [93], disaster preparedness, and reduced psychological harm [67].

## 3. Adaptation

Natural disasters are known to be accidental and to follow a predictable course after their manifestation; as a result, they are typically associated with better recovery. In contrast, human-induced or technological disasters are typically associated with a more complex and painful recovery, especially when those disasters are perceived to be preventable and avoidable. Climate change manifests itself through natural phenomena and disasters, but they are likely to be perceived as human-caused events, thus making it harder to accept and move on. These considerations play a crucial role in perceived responsibility and accountability and, therefore, in adaptation motivations [45,94].

Adaptation is about processing information and how to practically prepare for extreme events and to a changing environment. When a situation seems controllable it usually provokes active coping and produces adaptive strategies, while, on the other hand, avoidance strategies are distanced from the problem helping to minimize the gravity of the phenomenon and prevent those affected from feeling responsible for it [11].

Adaptation to climate change requires from humans a frequent switch between different levels of perspective: macro (DNA, resources, possibilities of species), meso (collectivity, ethnic realities, mass and psychosocial phenomena), and micro (psychological adaptation, family and individual). Given the complexity of the events and the number of variables involved, it is useful to integrate any explanation of adaptation to climate change with broader concepts from biology of species. Climate adaptation is context-dependent [94], it is linked to the type of threat and on what is damaged in the abiotic environment where the species live [95,96], but a clear comprehensive model of how different species might respond is still lacking [4,97,98], since extreme climate events and biological response have a nonlinearity dependency [99,100]. Extreme climate events affect the allostatic overload (i.e., the energy cost to survive) of an organism but the seriousness of the impact depends on the organism itself, and they generate hysteresis and genetic changes in species and in ecosystem functioning [101] that may be temporary or localized, but also long-lasting or leading to a regime shift.

Intergovernmental Panel on Climate Change (IPCC) defines adaptation as a “process of adjustment to actual or expected climate and its effects” [85]. There are three ways of adapting to climate change: genetics, behaviour, and migration, depending on intrinsic characteristics, such as genetic architecture, behavioural flexibility [102,103], life history, demographic factors, and dispersal capacities. However, genome and migration can be considered a single endogenously interrelated system, since successful adaptation has been associated with genes involved in exploration and dispersal capacity [104]. Genetic evolutionary properties and/or behavioural adaptation factors allow species to resist in their current geographic range, while dispersal potential and migration ability allow adaptation in different geographic conditions [9], since it improves the access to resources [104,105,106,107,108,109,110]. For humans, this phenomenon is called “ecomigration” and can also result in displacement and relocation, leading to “environmental refugees”, with growing concerns from international organizations such as the United Nations High Commissioner for Refugees (UNHCR) [109,111,112].

An alternative mechanism to mitigate the impact of extreme climate events are non-genetic answers, a mechanism known as “plastic rescue” [113]: phenotypic plasticity enables a range of phenotypes faster and without genetic variations, leading to modifications in morphology, behaviour, and physiology of living beings [4,16,96,114,115]. However, plastic responses could be effectively adaptive if there are genetic correlations between plastic responses under extreme and non-extreme conditions, and the phenotype changes smoothly with the environment [116]. In humans, the mind must be considered adaptive plasticity: it is a highly integrated, coherent, sentient, and pro-active type of information, and it may produce technologies that provide an advantage in a complex, changing environment [117,118], thus modulating human responses, both emotional and behavioural, at an individual and a collective level, eventually reinforcing resilience and adaptation. The role of technology became progressively more relevant in human existence, and it can be now considered the main phenotypic adaptation for human behaviour. With regard to climate change, it is either perceived to be part of the problem or part of the solution. Nevertheless, technological solutions are expected to play a key role, since they are the first tools to deal with the climate crisis (i.e., change or diversify means of support and energy, build sustainable cities, manage waste, and pollution). However, technological means are useless when leaderships are focused on maintaining power and control on resources, or they are too weak, or they have to deal with other threats.

## 4. Emergent Behaviours, Emotional Regulation, and Mass Effect Phenomena

In an adaptation effort, human collective behaviours may emerge when events are perceived as overwhelming or when the control in the use of information technology is lacking. These behaviours have also been called “emergent behaviours”, because they are not previously known or observed, and they arise in complex systems from the interaction of different parts, none of which displayed that behaviour individually. They can be either beneficial or potentially harmful.

Climate change information has different influences on individuals’ psychological believes [45,82,119,120,121], and the kind of climate change events is linked to different emotional regulation styles [46]. Sometimes, people may be distressed about climate change without knowing it, sensing only a vague unease about the changes happening around them [120]. The direct experience of repeated extreme climatic events on one’s territory can trigger a stronger reaction because the threat is perceived as concrete and close [7], whereas climate change is more abstract and distant if it is not directly witnessed [122]; therefore, a reduced “psychological distance” is associated with a higher level of concern and more pro-environmental intentions that lead to better adaptation. Nonetheless, predictions of future climate catastrophes and repeated exposure to climate information can stimulate constant vigilance, uncertainty, feelings of hopelessness and powerlessness, bad future expectation, and fears about the high cost of choices that have to be made [7], with a rise in the use of counterproductive emotional regulation strategies that may cause the developing of mental (i.e., emotional) disorders [46]. Strategies focusing on emotional regulation (rather than the problem) tend to illustrate passive coping, i.e., the feeling of helplessness to deal with the stressor, thus relying on others to solve the situation [123]. People may display apathy, numbness, until “total inertia” [13,82]: the increasing psychological distance between the self and complex tasks can increase tolerance and concealing, thus reducing the negative feeling of difficulty and avoiding climate-change-induced emotional challenge, but also leading to passive acceptance and acting non-defensively, a sort of paralysis in the face of the size of the problem, with disbelief, unfocused terror, overreaction to news and science and even aggression, slowing down or rejecting timely actions and survival choices [46,82,124,125,126], with a general worsening in climate change impacts [3,127], sometimes presumed to be inevitable [67] in a retrogressive “psychological isolation” [128,129] (a similar psychic phenomenon has already been described in Italy in the first half of the 20^th^ century and labelled as the “crisis of presence” [130]). This inner conflict between the need to react to danger and the need to protect the mind from being overwhelmed can result in a wait-and-see attitude, called “resilience paradox” [67]. Even if the majority of people are sufficiently aware of climate change and its future consequences, their behaviour is rather ineffective [131]. Furthermore, when danger messages are too similar and too frequent, they are frequently ignored [13,14]. People may also express scepticism and denial [85,128,132], and even paradoxical and non-adaptive collective behaviours [13,15], such as communities at risk that reduce their intentions to undertake pro-environmental behaviours [15,45] or elicit general authoritarian responses [133]. For instance, in a balance between the attachment for the land and the grief for the new changes, Arctic native communities who have to be relocated due to climate change may object because of a history of being moved against their wishes [134], and U.S. coastal homeowners may not significantly protect their homes despite the experienced increased frequency and intensity of tropical storms and sea level rise [135]. On the other hand, engaging more in adjusting allows people to focus on accessing emotional challenges and take a problem-solving approach [13,46,124,125,136,137,138].

Efficient communication [13] and correct information are fundamental to promote awareness and to ensure a quick response [139], starting from educating the young generations; opinions must be accompanied by skills to explore other alternatives without ending up as fake news [140,141]. Indeed, the more the message is specific and concrete, the more it is efficient. The Internet in particular has changed the way people deal with collective concerns, highlighting fears for non-immediate threats [142]. However, vague catastrophic previsions in the medium-term should be avoided, since they lead to the loss of trust. Managing the rise and the regulation of negative emotions is a part of all mass communicative phenomena. Mass aggregation and communication can create a collective way of thinking that we can imagine as a “human magnetic field”. In fact, in strongly interconnected systems, single units tend to display tight group behaviour [18]: social pressure and the opinion of peers can play an important role in individuals’ decision-making process [7]. Emotionally driven individuals behave more similar to ferromagnetic particles: they are more oriented towards the quantity of information [18] rather than select by the quality and lean towards scientifically oriented choices as they would do as individuals [143]. When a certain level of complexity has been reached, the effectiveness of this complexity starts to decline: energy causes complexity to grow, and higher complexity needs more energy in an exponential trendline, an inevitable energy–complexity spiral [144]. This may eventually lead people to confusion and dissatisfaction that, in turn, requires additional energy to be controlled. We are now witnessing a similar circumstance for the COVID-19 pandemic, forecasting what is expected to happen regarding the worsening of climate.

## 5. Mental Maladjustment and Socioeconomic Processes

The World Health Organization has defined climate change as one of the greatest health threats of the 21st century [145] and an increase in climate-change-related events will entail higher risks to human health and survival, also exacerbating the already increasing incidence of non-communicable diseases NCDs [146,147] (i.e., chronic diseases of long duration and the result of a combination of genetic, physiological, environmental, and behavioural factors) that include mental disorders [148]. The impact of climate change on people’s physical, mental, and community health can arise both directly and indirectly. The direct effects on mental health may occur rapidly, usually from extreme weather events and natural disasters, or gradually as slowly progressive but not necessarily life-threatening (e.g., changing temperature and rising sea levels). The indirect effects can be caused by poor physical health (which is associated with mental wellbeing), by environmental risk factors (such as smoke, pollen density, dust, plant disease, infestations, availability of water, water disease, living in urban slums, loss of sense of place), through the social environment (their impact on human activities), via adaptation and mitigation (e.g., travel by alternative means, availability of air conditioning) [56,121,149]. Moreover, climate change may have an impact on individuals and communities not directly affected by it, including emotional and affective responses, which can arise when observing climate change effects worldwide, viewing images of environmental degradation or human suffering in the media, questions of lifestyle or purchasing choices, usually being influenced by personal values, beliefs, and experience, and by social representations of climate change and its impacts [45,150,151].

Mental health effects of climate change range from minimal stress to clinical disorders, and they can be classified as acute or chronic, bearing in mind that some consequences may overlap. Acute effects are usually consequences of extreme and powerful weather events or natural disasters (such as wildfires, tornadoes, hurricanes, storms, floods, and droughts) that may occur with little or no warning, involving loss of life, resources, social support, and social network. Generally, such reactions can be diagnosed as acute stress disorders if they begin within four weeks after a disaster or, if longer, as post-traumatic stress disorders (PTSD), depression, and anxiety. Many exposed people exhibit hostility, paranoia [56,152], physiological hyperarousal, chronic dissociation, detachment, cognitive symptoms, poor quality of sleep, increased domestic violence, alcohol, substance abuse [56], psychosomatic disorders, and suicidal thoughts [42,56,109,153]. Oppressive heat waves may induce exhaustion [154], increased suicide rates [155], higher rates of self-harm, increased mortality and morbidity risks amongst psychiatric patients [156], as well an increased utilization of mental health services [149]. Chronic effects result from long-term changes in climate. In addition to PTSD, depression, and anxiety, it is possible to detect the presence of post-disaster adjustment, loss of identity, loss of autonomy and control, and many emotions such as fear, anger, helplessness, resignation, exhaustion. In addition, there are large-scale social and community effects, even taking the form of violence, conflicts over scarce resources, displacement, and migration [56,157,158,159]. Effects can be delayed and may also persist over several years affecting communities in broader areas as well [89]. Children can also be affected, showing posttraumatic reactive phenomena, such as anxiety disorders and panic attacks, sleep problems, adjustment disorder acute stress reactions, compulsively repetitive play, re-experiencing fears, and psychotic disorders [63].

In some papers, the association between climatic events and mental disorders has been labelled with new terms as eco-anxiety, eco-guilty, ecoparalysis, ecological grief, and solastalgia. Psychotherapists are pioneering a new field of treatment termed “ecopsychology”. “Ecoanxiety” can be described as anxiety related to a changing and uncertain environment and becoming overwhelmed by the sheer scale and complexity of the problem faced [56,160,161]. “Ecoparalysis” refers to apathy and disengagement with reality due to the inability to meaningfully respond to the climatic and ecological challenges, in contrast to expressions of avoidance [161]. *“*Ecological grief” is the grief associated with physical ecological losses (land, ecosystem, and species) and attendant ways of life and culture, or the grief associated with disruptions to environmental knowledge systems and resulting feelings of loss of identity, or the grief associated with anticipated future losses of place, land, species, and culture, due to acute or chronic environmental changes [162]. The term *“*solastalgia” refers to the pain or sickness caused by the loss or lack of solace from one’s home and territory, with the awareness that the living and loved place is in danger, leading to the fading of the sense of belonging (identity) to that particular place and a feeling of distress (psychological desolation) about its transformation [163,164]. In contrast to “somaterratic illnesses” (soma = body, terratic = Earth-related) that threaten physical wellbeing and are caused mainly by living in ecosystems that have been contaminated by pollutants and toxins, these new concepts can be synthesized as a form of “psychoerratic illnesses”, defined as an Earth-related mental illness where people’s mental wellbeing (psyche) is threatened by the severing of “healthy” links between themselves and their home/territory [165].

It is worth noting that there are no specific references or mention of mental disorders related to climate change in DSM 5 [166] and ICD 10 [167], and they are not expected in ICD 11.

Another topic that gained attention due to climate change as “global warming” is the relation between increased average surface temperature and violence. An increase in environmental temperature can impair the central nervous system and, therefore, psycho-physiological functions by affecting bio-chemicals (e.g., by altering the production of serotonin and dopamine) [156] or by disrupting the homeostasis of thermoregulation [168]. This may cause a decrease in awareness and self-regulation leading to increased feelings of hostility and aggression and a negative effect on cognitive functioning, possibly reducing the ability to resolve conflicts without recourse to violence [169]. Cities with hotter climates were more violent than cooler cities and the increase in heat-related violence was greater in summers during the hottest years [170]. Of course, the relation between climate change and conflicts can be also linked to the scarcity of resources, reduction in food production, increase in food prices, migration, and changes in the political economy of energy resources: these factors may lead to changes in the geographical distribution of populations and, consequently, in interpersonal and intergroup relations, leading to economic disruption and undermining human security, with relevant judicial disputes concerning access to diminishing resources and exacerbation of socio-economic disparities and intergroup conflicts [45,171,172]. Nevertheless, the escalation in the risk of violence and conflicts has been linked to an increase in temperature independently from other factors, noting that it is a global phenomenon not restricted to particular territories, such as within Africa, and neither to agricultural areas nor provinces that experience fluctuations in agricultural output. However, there is no proven evidence of a clear causal mechanism demonstrating an empirical relationship between climate change and violent conflicts [173]. Studies and calculations cannot predict such consequences with precision, and not all studies found a correlation [174,175].

**Figure 2:**
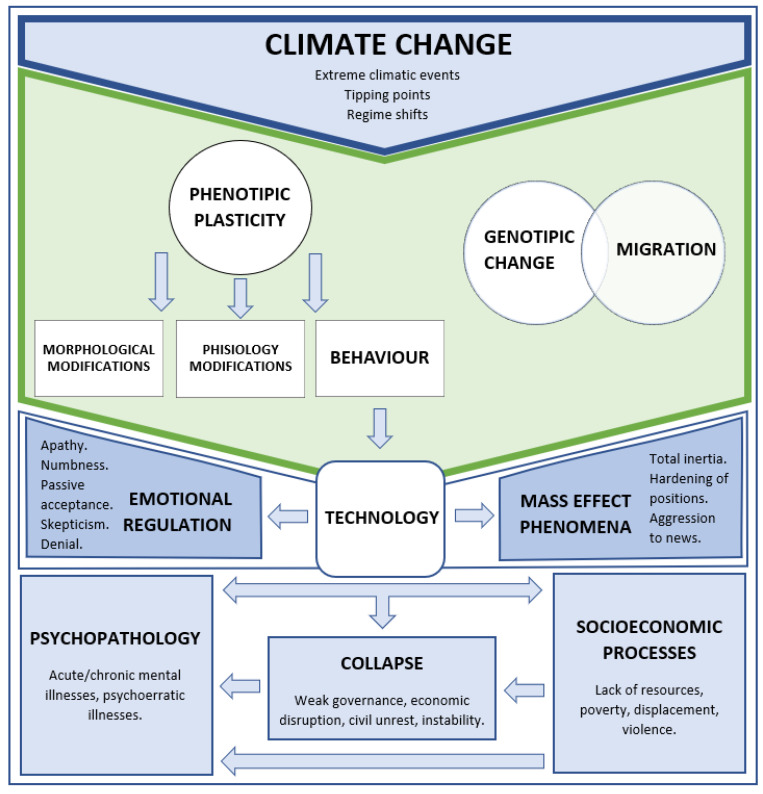
Conceptual framework on the relationships between climate change and human responses.

In short, climate change can be an important potential catalyst for the collapse of complex societies in general (Figure 2), not just because of climatological events per se, but also due to societal vulnerability to its consequences (e.g., a succession of severe natural disasters) in association with other societal stressors and risk factors, such as poverty, income inequality, weak governance, and a pre-existing history of conflicts [176]. That is why it is commonly conceptualized as a “threat multiplier”. Weak countries, poor countries, and those with the lowest levels of democracy tend to present a higher probability of instability [159], and they are often those also sharing environmental burdens, pollution, and the risk of climate change with less capacity for adaptation [177], and with a vulnerable socio-economic system leading to financial loss [56,178]. Thus, the increase in the disparity may not only be within nations but also between nations. In a domino effect scenario, climate change may undermine the capacity of states to protect people against natural disasters and the effects of environmental change, failing to promote human security and peace, leading people to lose confidence and trust in civil institutions, amplifying already existing social tensions and stressors, eventually resulting in civil unrest and ethnic clashes. Some groups may engage in conflict against the state, threatening the integrity of nations, causing a challenge to geopolitical stability and destabilizes conflict-prone regions, also triggering migration flows, with an impact on geographically bordering countries [76,133,176]. These change-related risks are acknowledged by major institutions (such as NATO, the US Department of Defense, and the European Union), and they are planning actions to confront this challenge [179,180].

## 6. Conclusions: Implications for the Future

Reactions to extreme weather events are similar to traumas from natural disasters, while long-term changes are more challenging. The pressure of climate change will possibly lead to new forms of mental distress, climate-related mental disorders (CRMD), and to new adaptations [181]. What seems relevant is not only the change itself but the broad difference between the “before” and “after” state, the speed of the disruption, and the time needed to reach a new stable state. Nonetheless, if changes in climate will keep an instable course with more frequent extreme events, we can assume that repeated failed efforts to adapt (at least for a while) will lead to a stratification of chronic distress and unsuccessful coping strategies. Mental adaptation to climate change is not just an individual but a collective adjustment, and resilience among people sometimes looks more like resistance to new configurations: pro-environmental behaviour seems to be very difficult to motivate and some of these maladaptive reactions seems to be deeply rooted in humans, making them less sensitive to change. Interestingly, it has been argued that people tend to construct their concept of what is environmentally normal on their experience, usually based on the natural world they encountered in childhood, and thus, may fall to recognize, over years and generations, the extent to which the environment has degraded, a process called “environmental generational amnesia”, highlighting the risk to adapt to the loss of nature [182]. In a more optimistic view, it may also become an opportunity to enhance our awareness of and action towards environmental protection, sustainability, and climate-sensitive health (both mental and physical) issues [45]. It is crucial to inform the population about the likely occurrence of extreme events linked to climate change, about the uncertainty of their duration and their effects, and how to actually prepare, even if it still has not been affected. Additionally, a “transgenerational psychological adaptation” must be addressed: in the future, will the older generation feel guilty for the irreversible damage of the environment? Additionally, the same time, will the younger generation feel angry and doomed because of negligence?

Moreover, it is important to consider the ethical and social justice implications of climate change as global inequalities and human rights are involved [183]. Transformative actions must rely on a long-term strategy starting from monitoring, evaluating, and reviewing adaptation planning and implementation. National-level coordination includes the provision of information concerning potential risks, assisting the state and local governments with direct action, providing resources for national development (agriculture, fisheries, health, ecosystem protection, among others), the protection of vulnerable groups, and the provision of financial resources [75]. Whilst most climate change impacts are indeed experienced locally (such as floods, reductions in crop yields, or spread of disease), they may provoke national and international consequences requiring coordinated actions and plans accessible to developing countries [43,90]. Communities affected by climate change must be supported not only by their respective governments, but also by the global community enabling them, when necessary, to move from their original place [184].

Governments must also address the climate impact of the corporate sector as some of the entities most responsible for global emissions in 40 years of neoliberal hegemony, taking into account its influence to drive policy changes and shape consumer preferences. An industrial transition towards a more sustainable economy must be a primary political intervention, since the economic dimension prevails in the corporations’ strategies.

Cross-sectional and interdisciplinary collaborations are needed, while care providers and mental health professionals must be aware and properly trained to support different levels of interventions, from first responding in the case of a natural disaster or a climate emergency to community-based interventions and fostering successful adaptation. The proposed framework is meant to offer a concise summary of the many perspectives involved in climate change as a contribution in helping researchers to contextualize their studies in a broader overview.

As human beings, we must recognize our innate affiliation with actual nature, the benefits of which cannot be replaced by the increasingly sophisticated and pervasive forms of technological nature [185]. An eco-sustainable way of thinking is not merely a matter of biological survival and adaptation, it has to do with the essence of humanity.

## Figures and Tables

**Table 1:** Timeline of the more relevant social and cultural crisis associated with changes in climate along human history.

Time Period	Location	Event
Around 10,000 BC	Middle East	Disappearance of the Natufian culture (end of the Last Glacial Period). Later, a new thermal optimum helped the Neolithic agricultural revolution.
After 3500 BC	Mesopotamia	Emergence of city-states.
Around 2150 BC	Egypt	Collapse of the Old Kingdom.
Around 2150 BC	Mesopotamia	Collapse of Akkadian empire.
Around 1700 BC	Egypt	Collapse of the Middle Kingdom. Later, invasion by the Asian Hyksos.
Around 1700 BC	India	Collapse of the Harappa civilization. Later, invasion of the Aryans.
5th century BC–2nd century AD	Roman Empire	Roman expansion during the 1st century BC (Roman Warm Period).
5th century AD–7th century AD	Roman Empire	Climate crisis during the collapse of the Roman Empire.
9th century AD	Mesoamerica	Crisis and collapse of the Mayan civilization.
Around 1275	North America	Abandoning of the Anasazi pueblos.
1347	Europe	The Black Death. End of Medieval Warm Period (around 1300).
Half of the 14th century	China	End of the Mongols’ power and the start of the Ming dynasty.
14–15th century	Greenland	Decline and disappearance of the Viking colonies.
1570–1630	Europe	Resurgence of epidemics in periods of adverse climate conditions and poor harvest.
16–17th century	Europe	Mass-social reactions linked to climate crises (e.g., peak of witch-hunts).

## Data Availability

Not applicable.
